# The effect of apigenin and chemotherapy combination treatments on apoptosis-related genes and proteins in acute leukaemia cell lines

**DOI:** 10.1038/s41598-022-11441-z

**Published:** 2022-05-25

**Authors:** Amani A. Mahbub, Christine L. Le Maitre, Neil A. Cross, Nicola Jordan-Mahy

**Affiliations:** 1 Laboratory Medicine Department, Faculty of Applied Medical Sciences, Umm Al‐Qura University, P.O. Box 715, Makkah, 21955 Saudi Arabia; 2 Biomolecular Sciences Research Centre, Sheffield Hallam University, The Owen Building, City Campus, Howard Street, Sheffield, S1 1WB UK

**Keywords:** Cancer, Cell biology, Drug discovery, Molecular medicine

## Abstract

Apigenin is a dietary polyphenol found abundantly in fruit and vegetables, which sensitizes leukaemia cells to topoisomerase inhibitor agents (e.g., etoposide), and alkylating agents (e.g., cyclophosphamide), reducing ATP levels and inducing apoptosis; whilst being protective to control haematopoietic stem cells. This study analysed the expression profiles of intrinsic and extrinsic apoptosis-related genes and proteins to help elucidate the mechanisms of action of apigenin when used in combination with etoposide or cyclophosphamide in lymphoid and myeloid leukaemia cell lines (Jurkat and THP-1). Expression of apoptosis-related genes were measured using a TaqMan® Human Apoptosis Array and the StepOne Plus RT-qPCR System, whilst apoptosis-related proteins were determined using a protein profiler™-human apoptosis array and the LI-COR Odyssey^R^ Infrared Imaging System. Apigenin when combined with etoposide or cyclophosphamide-induced apoptosis via the mitochondrial pathway, increasing the expression of pro-apoptotic cytochrome *c*, SMAC/DIABLO, and HTRA2/OMI, which promoted caspase-9 and -3 activation. Targeting anti-apoptotic and/or pro-apoptotic members of the apoptotic pathways is a promising strategy to induce cancer cell death and improve sensitivity to chemotherapy agents. Here the apoptotic pathways induced by apigenin in combination with etoposide or cyclophosphamide were identified within human leukaemia cell lines, such applications could provide combination therapies for the treatment of leukaemia.

## Introduction

Based on data from the International Agency for Research on Cancer, leukaemia is the tenth most common cause of cancer deaths worldwide^1^. Leukaemia is a group of life-threatening malignant disorders of the blood and bone marrow, which affects all blood cell lineages and is associated with an increase in the growth rate of blood-forming cells, caused by a failure of programmed cell death pathways and a reduced differentiation of haematopoietic cells^2–4^. Apoptosis plays a crucial role in determining and regulating cellular development, homeostasis, and both physiological and pathological processes^5^. Apoptosis has two core pathways: the intrinsic and the extrinsic pathway^5^. Dysregulation of programmed cell death via apoptosis in leukaemia is common due to abnormal expression or mutation of one or more apoptotic gene, resulting in the survival of malignant cells over their normal counterparts^6–8^.

Acute leukaemia is associated with a decrease in cell sensitivity to pro-apoptotic signals by overexpression of BCL-2 family anti-apoptotic proteins such as BCL2, BCLX, and MCL1^3,9,10^; and/or with low levels of pro-apoptotic members such as BAX^11^. This means leukaemic cells can evade apoptosis, and this can lead to chemotherapy resistance, and relapse associated with shorter disease-free, or overall survival for patients with acute leukaemia’s^3,8,9,11^. The standard front-line therapeutic approach for acute leukaemia patients is chemotherapy, which consists of multi-agent chemotherapy regimens^12–16^. Unfortunately, the multi-agent chemotherapy regimens have a number of off-target effects, which include pancreatitis, coagulopathy, and hepatotoxicity^14,15^ and the development of chemoresistance, which contributes to disease relapse, and ultimately patient death^14–17^. It is reported that this is particularly challenging in the treatment of childhood acute lymphocytic leukaemia (ALL), where there is a risk of relapse in 20–30% of children^18^.

Currently, one of the most promising anti-leukaemia strategies is to design targeted therapies with lower toxicities, which reverse chemotherapy-resistance, and improve acute leukaemia patient survival^3,8^. Such therapies would induce apoptotic cell death in leukaemia cells, with no or limited collateral damage to normal haematopoietic progenitor cells; and less likely to encounter many of the resistance mechanisms that have been associated with conventional agents^3,8,19^. Indeed, Rodriguez et al*.*, 2015 reported that intermediates of apoptosis pathways might be excellent candidates for molecular targeting for cancer treatment^20^. For this reason, it is worth identifying new drugs targeting apoptotic molecules that are abnormally expressed or dysregulated in leukaemic cells; and to restore the sensitivity of leukaemic cells to apoptotic stimuli.

Polyphenols represent a unique class of phytochemicals that possess excellent antioxidant, and anti-inflammatory properties, and modulate cell signalling pathways leading to anti-cancer effects^21–23^. Epidemiological data has shown that diets rich in polyphenols significantly improve the quality of life and survival rates of patients with a range of chronic diseases, including cancer^21–24^. Recently, Montané et al., 2020 reported that using polyphenols, as an alternative to anti-cancer drugs is a very promising approach, because they minimize or suppress the adverse effects of standard therapies. One such polyphenol is apigenin, it is found at high levels in herbs, fruits, and vegetables, such as oregano, parsley, celery, celeriac, carrots, and chamomile tea^23,24^.

At present, there is evidence suggesting that apigenin has an anti-cancer effect on 14 different cancers including breast^25–30^; prostate^31–33^; colon^34–38^; bladder^39,40^; cervical^41,42^; lung^43^; oesophageal^44^; liver^45^; pancreatic^46^; and bone cancer^47^; as well as glioblastoma^48^; melanoma^49^; lymphoma^50^; and leukaemia^51–55^. Within leukaemia cell lines, apigenin suppresses cell proliferation, induces cell cycle arrest, and apoptosis of leukaemia cells, in vitro^55^. Interestingly, apigenin has no anti-proliferative effect in normal cells, suggesting its potential as an anti-cancer compound, with no toxicity to normal cells^53,55^. Hussain et al., 2010 also reported that apigenin has low intrinsic toxicity and limited mutagenic properties, compared to other structurally related flavonoids^50^.

Most of these studies suggest that apigenin can induce apoptosis in cancers mainly via a mitochondrial-dependent mechanism^26,49–54^. Apigenin-mediated downregulation of BCL-2, causes the release of cytochrome *c* from the mitochondria to the cytosol, which leads to the activation of caspase-9 and caspase-3^26,49–54^. Likewise, previous research has shown that apigenin induces caspase 3 activity and apoptosis in various types of lymphoid (Jurkat, CCRF-CEM, MOLT-3, U937) and myeloid (HL60, THP-1, K562, KG1a) leukaemia cell lines with minimum effect on non-tumour blood progenitor cells (CD34^+^)^55^. Notably, apigenin produced a greater toxicity on lymphoid than myeloid leukaemia cells^55^.

Furthermore, the use of combination treatments can improve the response to chemotherapy, clinical outcome, and patient survival rates^22,56^. Montané et al., 2020 suggested that chemotherapy agents could be combined with polyphenols, as polyphenols could enhance the efficacy of chemotherapy agents and decrease the development of drug resistance, toxicity, and side effects on human health^23^. There have been promising results in in vitro and in vivo studies using combination treatments of apigenin and chemotherapeutic agents, this has opened a new avenue of drug discovery for cancer. Recent studies showed that apigenin can synergistically enhance the anti-cancer activity of 5-fluorouracil in the treatment of breast cancer cell lines (MDA-MB-453)^57^ and head and neck cancer cell lines (SCC25 and A431)^58^; paclitaxel in the treatment of cervical cancer cell lines (HeLa)^59^; ABT-263 in the treatment of colon cancer cell lines (HTC-116)^60^; and cisplatin in the treatment of lung cancer cell lines (A549)^61^. In a recent study by Huang et al., 2020, they demonstrated apigenin synergistically enhanced the action of the BTK inhibitor abivertinib, and this action was associated with an apigenin-mediated downregulation of BCLX by the suppression of the PI3K/p-AKT pathway in diffuse large B-cell lymphoma cell lines (U2932 and OCI-LY10) at 24 h^62^. All these previous studies suggest that apigenin has great potential to improve cancer therapy.

This is supported by our previous work, which demonstrated apigenin could synergistically enhance the action of topoisomerase inhibitor agents (e.g., etoposide), and alkylating agents (e.g., cyclophosphamide) reducing ATP levels, and inducing apoptosis (caspase 8, 9 and 3 induction and morphological evidence of apoptosis) in both lymphoid and myeloid leukaemia cell lines; whilst protecting normal hematopoietic stem cells (CD133^+^HSC and CD34^+^HSC)^63,64^. The synergistic effect of these combination treatments was shown to be dependent on the modulation of glutathione (GSH) levels, caspase cascades (8, 9 and 3), and DNA damage ^63,64^. Importantly, a reduction of GSH was strongly linked with sensitising leukaemia cells to the pro-apoptotic effects of polyphenols like apigenin when used in combination with etoposide^63^ or cyclophosphamide^64^.

However, the exact molecular mechanisms underlying pro-apoptotic effects associated with apigenin in combination with these chemotherapies have not been fully elucidated. The expression of genes that regulate apoptotic cell death plays an essential role in determining the sensitivity of cancer cells to chemotherapy^65^. For this reason, this study aims to analyse the expression profiles of intrinsic and extrinsic apoptosis-related genes and proteins, to help elucidate the molecular mechanisms of action of apigenin in combination with chemotherapy agents (etoposide and cyclophosphamide) on signalling molecules that play important roles in regulating programmed cell death of acute lymphoid and myeloid leukaemia.

## Materials and methods

### Experimental design

Two human leukaemia cell lines were selected to represent lymphoid and myeloid lineages (Jurkat and THP-1). Apigenin has been previously shown to synergistically induce apoptosis when combined with etoposide and cyclophosphamide within these cells^63,64^. However, the apoptotic pathways induced by such treatments remain to be elucidated. To investigate apoptosis related gene and protein regulation induced by apigenin alone or in combination with chemotherapy agents, Jurkat and THP-1 cells were treated with the lowest significant doses (LSD) to induce apoptosis selected from our previous studies^55,63,64^. Following treatment for 24 h a qPCR based TaqMan™ Array of Human Cellular Apoptosis Pathway was utilised to investigate gene expression regulation of 92 apoptosis-related genes (Supplementary Table S1). A subsequent analysis was made of 35 apoptosis-related proteins (Supplementary Table S2) using the Proteome Profiler™ -Human Apoptosis Array Kit. These analyses were utilised to investigate potential gene and protein regulation by apigenin alone and in combination with chemotherapy agents.

### Leukaemia cell line culture

Two human leukaemia cell lines were selected for this study: one acute myeloid leukaemia cell line [THP-1 (monocytic leukaemia)] (ATCC, TIB- 202) and one acute lymphoid leukaemia cell line [Jurkat (peripheral blood T cell leukaemia)] (ATCC, TIB-152). Cells were cultured as described in Mahbub et al., 2013^55^, and incubated under standard cell culture conditions at 37 °C with a 5% CO_2_ atmosphere. Furthermore, cells were tested for mycoplasma contamination using the MycoAlert™ mycoplasma detection kit (Lonza, LT07-318) and were negative throughout all experiments.

### Treatment regimes

Cells were seeded at 1 × 10^6^ per well in 12-well plates and treated with the lowest significant doses (LSDs) of apigenin (AP) (Sigma Aldrich, 10798) at 10 µM and 50 µM, etoposide (ETP) (Sigma Aldrich, E1383) at 0.01 µM and cyclophosphamide (CYCLO) (Sigma Aldrich, PHR1404) at 2 µM and 10 µM, alone or in combination; along with a vehicle control for 24 h. The therapeutic agents: AP, ETP, and CYCLO were prepared as described previously^55,63,64^. LSDs capable of inducing apoptosis were selected based on our earlier published data^55,63,64^ which caused a 10% to 20% increase in caspase-3 activity and induced apoptosis in THP-1 and Jurkat cells, when compared to the vehicle control.

### Gene expression analysis using a TaqMan® Human Apoptosis Array based on the RT-qPCR reaction

Treated cells were collected and the RNA was extracted using a PureLink™ RNA Mini Kit (Thermofisher Scientific-Invitrogen, 12183018A) following the manufacturer’s instructions. The purified RNA was then transcribed to cDNA using High-Capacity RNA-to-cDNA™ Kit (Thermofisher Scientific- Applied Biosystems, 4387406) according to the manufacturer’s instructions.

Following preparation of the cDNA, a 10 µl of PCR reaction mix was prepared with the 5 µl of cDNA and 5 µl of TaqMan™ Fast Advanced Master Mix (Thermofisher Scientific- Applied Biosystems, 4444557) following the manufacturer’s instructions and loaded onto the TaqMan Array Human Apoptosis Fast 96-Well Plate Pathway (Thermofisher Scientific-Applied Biosystems, 4418762). Next, the plate was loaded into the StepOnePlus™ Real-Time PCR system (Applied Biosystems, Sheffield Hallam University, UK). Thermal cycling conditions consisted of a hold step for 20 s at 95 °C, and then amplification for 40 cycles starting with an initial denaturation step at 95 °C for 3 s and an annealing/extending step at 60 °C for 30 s. The relative gene expression was determined using the 2^*−∆∆CT*^ method^66^. Normalizing against the average housekeeping genes of 4 endogenous control genes: transferrin receptor (TFRC), glyceraldehyde-3-phosphate dehydrogenase (GAPDH), hypoxanthine phosphoribosyltransferase 1 (HPRT1), and Beta glucuronidase (GUSB) (Supplementary Table S1); and untreated controls. Three arrays were processed for each treatment in THP-1 and Jurkat cell lines, in three technical experiments. The results are expressed as medians and ranges, of three independent repeats.

### Protein expression analysis using a proteome profiler™: human apoptosis array

Treated cells were collected and washed twice with PBS. Cells were lysed with CelLytic M (Sigma Aldrich, C2978, 10 ml/g) supplemented with a protease inhibitor cocktail (Sigma Aldrich, P2714, 1:100) as described in the manufacturer’s instructions. The total protein content was quantified using the Pierce™ BCA protein assay kit (Thermofisher Scientific, 23227), following the manufacturer's instructions. The expression of 35 apoptosis-related proteins was assessed using the Proteome Profiler™—Human Apoptosis Array Kit (Biotechne—R&D Systems, ARY009) (Supplementary Table S2). In brief, the Human Apoptosis Proteome Profiler™ array was composed of a nitrocellulose membrane with duplicate spots for each apoptosis-related protein. Protein expression analysis was performed in duplicate on arrays (each array contained two spots for each protein analysed) for duplicate technical repeats for each treatment in each type of cell, thus a total of 4 repeats per protein per treatment group. All analyses were performed according to the manufacturer's instructions.

Cell lysates were analysed using the proteome profiler human apoptosis antibody array kit (Biotechne—R&D Systems, ARY009) in accordance with the manufacturer’s instructions, utilising 300 µg of cell lysate per membrane. Thereafter, the array membranes were scanned using the LI-COR Odyssey^R^ Infrared Imaging System (LI-COR). The pixel density of each duplicated array spot was quantified using Image Studio™ Software version 5.2 (LI-COR). Following the manufacturer’s instructions (Biotechne—R&D Systems, ARY009), the mean pixel density of each duplicated protein spot was determined and subtracted from the mean density of the background/reference spots. Reference spots are included in each array to align the transparency overlay template and to demonstrate that the array has been incubated with IRDye® 800CW Streptavidin (LI-COR Biosciences UK, 926-32230) during the assay procedure. The results were expressed as a median and range of two independent experiments.

### Statistical analysis

The median and range was determined for each assay. Stats Direct software (Stats Direct Ltd, Altrincham, UK) was used to determine if the data followed a normal distribution using a Shapiro–Wilk test. As data was identified as non-parametric, a Kruskal–Wallis with a Conover- Inman post-hoc test was used to determine statistical significance. Significance was set at P ≤ 0.05. Statistical significance of individual agents was determined firstly in comparison to the vehicle control (VC) (Supplementary Tables S3–S6). The statistical significance of combined apigenin and chemotherapy treatments was determined in comparison to the vehicle control and the individual treatments alone (Supplementary Tables S3–S6) to determine potential synergistic or antagonistic responses (see below). The results were shown as relative fold change in gene expression/protein levels, with the vehicle control being set at 1.

#### Individual treatment effects

A significant increase or decrease following individual treatments on apoptotic gene and protein expression was determined when compared to the vehicle control (VC) (P ≤ 0.05) (*). A significant increase is shown in Table 1 as an upward arrow in green (↑), whilst a significant decrease is shown as a downward arrow in red (↓). Where there were no significant changes in gene/protein expression compared to the vehicle control (VC); this was represented by a dash (**–**).

**Table 1:**
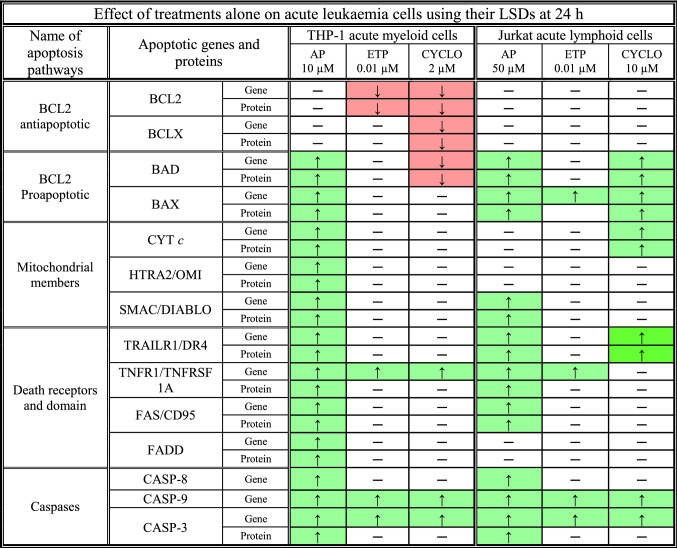
A summary of the effects of individual treatments: apigenin (AP), etoposide (ETP), and cyclophosphamide (CYCLO) on the expression of apoptosis-related genes and proteins in acute myeloid (THP-1) and lymphoid (Jurkat) leukaemia cell lines; using their lowest significant doses (LSDs) that induce apoptosis (previously determined in Mahbub et al., 2013, 2015, 2019) for 24 h treatment.

A Kruskal Wallis with an Inman post-hoc test was used to determine statistical significance and P ≤ 0.05. The individual treatment effects were determined as described in Statistical analysis.

#### Combination treatments effects:

The effect of combination treatments on apoptotic gene and protein expression were classified either as:

- **Synergistic—**here there is a significant increase or decrease in gene/protein expression compared to the vehicle control (VC) and drugs alone (P ≤ 0.05) (*). Synergistic increases in gene/protein expression are represented in the bar charts in dark green, whilst a synergistic decrease is represented in the bar charts in dark red.
- **Non-interactive, but with significant effect—**here there is a significant increase or decrease in gene/protein expression compared to the vehicle control (VC) and/or one of the individual drugs alone (P ≤ 0.05) (*). The significant increase in gene/protein expression is represented in the bar charts in light green, whilst a significant decrease is represented in the bar charts in pink.
- **No effect**—here there is no significant difference in gene/protein expression compared to the vehicle control (VC) and/or one of the individual drugs. This effect is represented in the bar charts in grey.

## Results

Fourteen apoptosis-related genes were significantly modulated and are discussed further here, these included: BAD, BAX, BCL2, BCLX, CYT c, SMAC/DIABLO, HTRA2/OMI, TRAILR1/DR4, TNFR1/TNFRSF1A, FAS/CD95, FADD, CASP-8, CASP-9, and CASP-3, no significant differences were observed in any of the remaining 78 apoptosis related genes (Supplementary Table S1) and thus are not discussed further here. Furthermore, 12 apoptosis-related proteins out of 35 investigated (Supplementary Table S2) were significantly modulated, which included: BAD, BAX, BCL2, BCLX, CYT c, SMAC/DIABLO, HTRA2/OMI, TRAILR1/DR4, TNFR1/TNFRSF1A, FAS/D95, FADD, and CASP-3.

### Effect of individual drug alone on apoptosis-related gene and protein expression in acute leukaemia cells

#### Apigenin

In THP-1 myeloid leukaemia cells, the use of the LSD for apigenin of 10 µM, caused a significant increase of pro-apoptosis-related genes and proteins (BAD, BAX, CYT *c*, HTRA2/OMI, SMAC/DIABLO, TRAILR1/DR4, TNFR1/TNFRSF1A, FAS/CD95, FADD, CASP-8, CASP-9 and CASP-3), when compared to the vehicle control (P ≤ 0.05). Whilst anti-apoptotic genes and proteins (BCL2 and BCLX) were unchanged (Table 1).

In contrast, Jurkat lymphoid leukaemia cells when treated with the LSD for apigenin (50 µM) for 24 h, displayed a significant increase in nine pro-apoptotic genes and/or proteins (BAD, BAX, SMAC/DIABLO, TRAILR1/DR4, TNFR1/TNFRSF1A, FAS/CD95, CASP-8, CASP-9 and CASP-3) compared to the vehicle control (P ≤ 0.05), with no changes seen in the remaining investigated genes and proteins (Table 1).

#### Etoposide

Treatment of THP-1 and Jurkat cells with the LSD of etoposide (0.01 µM) for 24 h significantly increased the expression of the pro-apoptotic genes: TNFR1/TNFRSF1A, CASP-9, and CASP-3 compared to the vehicle control (P ≤ 0.05) (Table 1), plus there was an increase of BAX expression in the Jurkat lymphoid leukaemia cells when compared to the vehicle control (P ≤ 0.05) (Table 1).

Etoposide treatment also caused a significant decrease of the anti-apoptotic factor BCL2 gene expression and protein levels in THP-1 myeloid leukaemia cells, when compared to the vehicle control (P ≤ 0.05) (Table 1). However, there was no significant effect on the remaining investigated apoptotic genes or proteins in both cell lines (Table 1).

#### Cyclophosphamide

Following treatment with the LSD of cyclophosphamide for THP-1 myeloid (2 µM) and Jurkat lymphoid (10 µM) leukaemia cells for 24 h, this caused differential effects on the apoptosis genes and proteins (Table 1). In THP-1 acute myeloid leukaemia cells, there was a significant decrease in the expression of BCL2, BCLX, and BAD genes and proteins; with a significant increase in TNFR1/TNFRSF1A, CASP-9, and CASP-3 gene expression, when compared to the vehicle control (P ≤ 0.05) (Table 1).

In Jurkat acute lymphoid leukaemia cells, there was a significant increase in the expression of BAD, BAX, and CYT *c* genes and proteins, as well as CASP-9 and CASP-3 genes when compared to the vehicle control (P ≤ 0.05) (Table 1). However, there were no significant effects on the remaining apoptotic genes or proteins in either cell line (Table 1).

### Effect of combination treatments on expression of BCL-2 family anti-apoptotic genes and proteins (BCL2 and BCLX)

**Figure 1:**
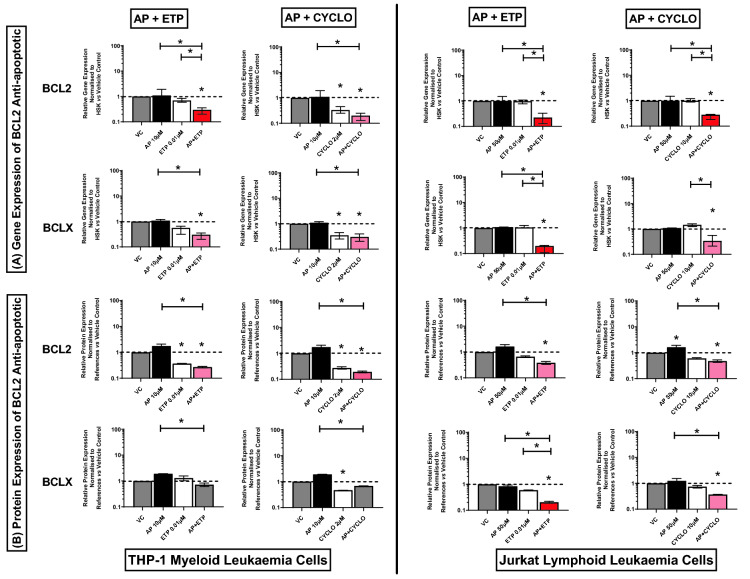
Effects of apigenin (AP) alone and in combination with etoposide (ETP) and cyclophosphamide (CYCLO) on expression of BCL2 and BCLX anti-apoptotic genes (**A**) and proteins (**B**) in acute myeloid (THP-1) and lymphoid (Jurkat) leukaemia cell lines when treated with their lowest significant doses (LSDs) that induce apoptosis (determined previously in Mahbub et al., 2013, 2015, 2019) for 24 h. Gene and protein data are expressed as medians and ranges. Results were considered statistically significant when P ≤ 0.05 (*). The combination treatment effects and colours were determined based on the statistical analysis described.

When apigenin was used in combination with etoposide, there was a synergistic decrease of BCL2 gene expression in both myeloid (THP-1) and lymphoid (Jurkat) leukaemia cells compared to both apigenin alone and etoposide treatment alone (Fig. 1A). Similarly, when apigenin was used in combination with cyclophosphamide there was a synergistic decrease on BCL2 gene expression in Jurkat lymphoid leukaemia cells only (Fig. 1A), when compared to the vehicle control and drugs alone, (P ≤ 0.05), with no further decrease seen in THP-1 cells when cyclophosphamide was combined with apigenin compared to cyclophosphamide alone (Fig. 1A). However, whilst protein expression for BCL2, was significantly decreased following apigenin and etoposide combination treatments compared to vehicle controls (P ≤ 0.05), in both THP-1 and Jurkat cells a synergistic response was not observed (Fig. 1B) (Supplementary Figs. S1, S2).

Apigenin when used in combination with etoposide was shown to synergistically decrease the expression of BCLX gene and protein levels in Jurkat lymphoid leukaemia cells, compared to the vehicle control and drugs alone (P ≤ 0.05) (Fig. 1A). Similarly, apigenin when used in combination with cyclophosphamide, also caused a significant decrease in the expression of BCLX gene and protein in Jurkat cells (Fig. 1A,B) (Supplementary Figs. S1, S2) when compared to the vehicle control and one of the individual treatments, but not both and thus failed to show synergistic responses (P ≤ 0.05). In contrast, in THP-1 myeloid cells, apigenin when combined with cyclophosphamide and etoposide caused a significant reduction in BCLX gene expression compared to apigenin treatment alone, but failed to result in a synergistic response (Fig. 1A), whilst protein levels remained unchanged (Fig. 1B) (Supplementary Figs. S1, S2).

Overall, Jurkat lymphoid cells were more susceptible to combination treatments, which caused a reduction in anti-apoptotic genes and proteins; whilst the THP-1 myeloid cells were unresponsive (Fig. 1A,B) (Supplementary Figs. S1, S2).

### Effect of combination treatments on expression of BCL-2 family pro-apoptotic genes and proteins (BAD and BAX)

**Figure 2:**
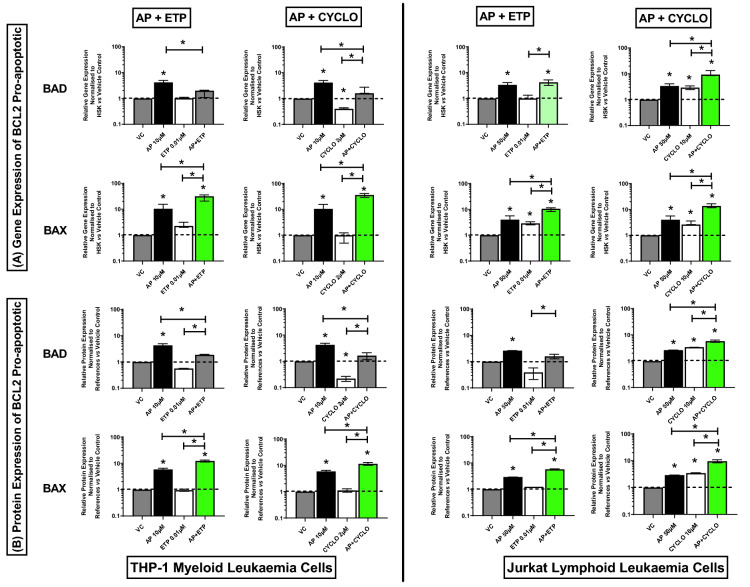
Effects of apigenin (AP) alone and in combination with etoposide (ETP) and cyclophosphamide (CYCLO) on expression of BAD and BAX pro-apoptotic genes (**A**) and proteins (**B**) in acute myeloid (THP-1) and lymphoid (Jurkat) leukaemia cell lines when treated with their lowest significant doses (LSDs) that induce apoptosis (determined previously in Mahbub et al., 2013, 2015, 2019) for 24 h. Gene and protein data are expressed as medians and ranges. Results were considered statistically significant when P ≤ 0.05 (*). The combination treatment effects and colours were determined based on the statistical analysis described in.

When apigenin was combined with chemotherapy agents in THP-1 cells the induction of BAD which was seen following treatment with apigenin alone, was inhibited in combination treatments (Fig. 2B). However, when Jurkat cells were treated with a combination of apigenin and cyclophosphamide there was a synergistic increase in the expression of BAD gene and protein levels compared to the vehicle controls and drugs alone, (P ≤ 0.05) (Fig. 2A,B) (Supplementary Figs. S1, S2). However, when apigenin was combined with etoposide in Jurkat cells there was a significant increase in BAD gene expression when compared to the vehicle controls and etoposide alone, but a synergistic response was not seen (P ≤ 0.05) (Fig. 2A); and protein expression was unchanged (Fig. 2A,B) (Supplementary Figs. S1, S2). Interestingly, all investigated combination treatments caused a synergistic increase in the expression of BAX gene and protein levels in both THP-1 and Jurkat cells when compared to the vehicle controls and drugs alone, (P ≤ 0.05) (Fig. 2A,B) (Supplementary Figs. S1, S2).

### Effect of combination treatments on expression of mitochondrial genes and proteins (CYT *c*, SMAC/DIABLO, HTRA2/OMI)

**Figure 3:**
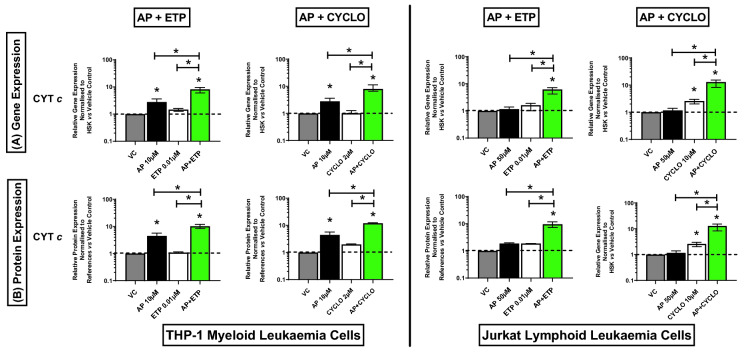
Effects of apigenin (AP) alone and in combination with etoposide (ETP) and cyclophosphamide (CYCLO) on expression of Cytochrome c (CYT *c*) mitochondrial gene (**A**) and protein (**B**) in acute myeloid (THP-1) and lymphoid (Jurkat) leukaemia cell lines when treated with their lowest significant doses (LSDs) that induce apoptosis (determined previously in Mahbub et al., 2013, 2015, 2019) for 24 h. Gene and protein data are expressed as medians and ranges. Results were considered statistically significant when P ≤ 0.05 (*). The combination treatment effects and colours were determined based on the statistical analysis described in.

**Figure 4:**
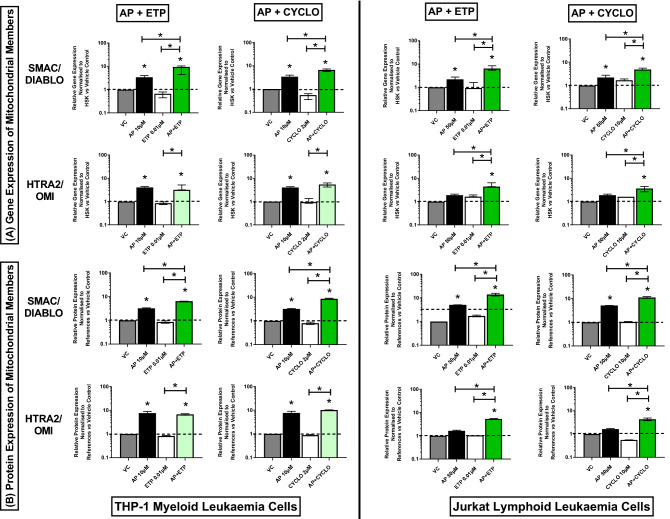
Effects of apigenin (AP) alone and in combination with etoposide (ETP) and cyclophosphamide (CYCLO) on expression of SMAC/DIABLO and HTRA2/OMI mitochondrial genes (**A**) and proteins (**B**) in acute myeloid (THP-1) and lymphoid (Jurkat) leukaemia cell lines when treated with their lowest significant doses (LSDs) that induce apoptosis (determined previously in Mahbub et al., 2013, 2015, 2019) for 24 h. Gene and protein data are expressed as medians and ranges. Results were considered statistically significant when P ≤ 0.05 (*). The combination treatment effects and colours were determined based on the statistical analysis described in.

In both acute myeloid and lymphoid leukaemia cells (THP-1 and Jurkat), all the investigated combination treatments synergistically increased the gene and protein expression of CYT *c* (Fig. 3A,B) (Supplementary Figs. S1, S2), and SMAC/DIABLO (Fig. 4A,B) (Supplementary Figs. S1, S2), when compared to the vehicle controls and drugs alone, (P ≤ 0.05). Similarly, there was a synergistic increase in the expression of HTRA2/OMI gene and protein in Jurkat lymphoid leukaemia cells when treated with all the combination treatments (P ≤ 0.05) (Fig. 4A,B). However, in THP-1 myeloid leukaemia cells, there was a significant increase in the expression of HTRA2/OMI gene and protein when compared to the vehicle controls and the chemotherapy drugs alone (P ≤ 0.05), but a synergistic response was not observed (Fig. 4A,B) (Supplementary Figs. S1, S2).

### Effect of combination treatments on expression of death receptors/domain genes and proteins (TRAILR1/DR4, TNFR1/TNFRSF1A, FAS/CD95, FADD)

**Figure 5:**
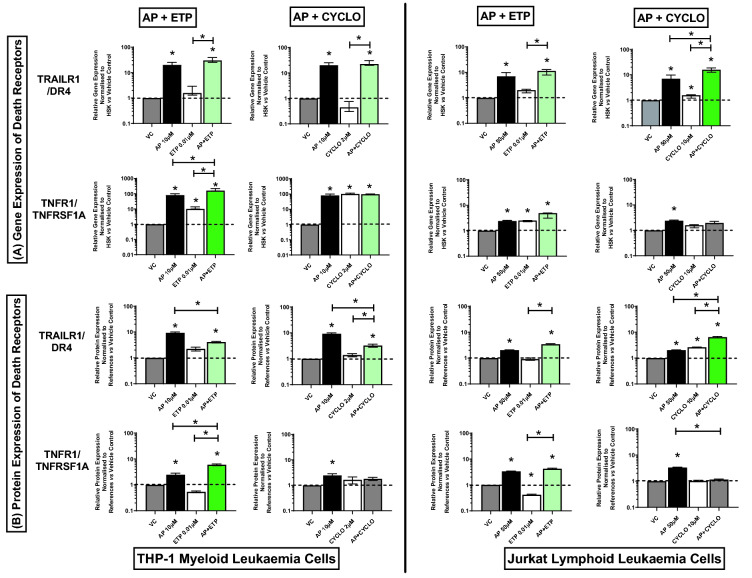
Effects of apigenin (AP) alone and in combination with etoposide (ETP) and cyclophosphamide (CYCLO) on expression of TRAILR1/DR4 and TNFR1/TNFRSF1A death receptors genes (**A**) and proteins (**B**) in acute myeloid (THP-1) and lymphoid (Jurkat) leukaemia cell lines when treated with their lowest significant doses (LSDs) that induce apoptosis (determined previously in Mahbub et al., 2013, 2015, 2019) for 24 h. Gene and protein data are expressed as medians and ranges. Results were considered statistically significant when P ≤0.05 (*). The combination treatment effects and colours were determined based on the statistical analysis as described in.

**Figure 6:**
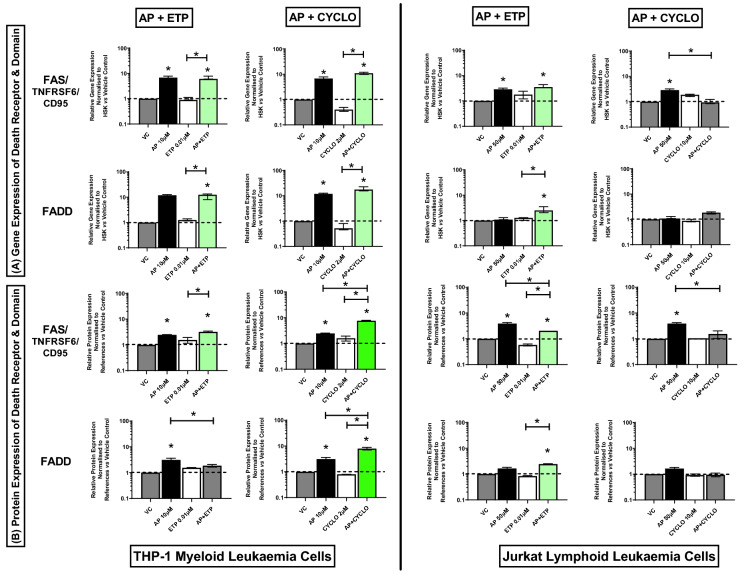
Effects of apigenin (AP) alone and in combination with etoposide (ETP) and cyclophosphamide (CYCLO) on expression of FAS/CD95 death receptor and FADD domain genes (**A**) and proteins (**B**) in acute myeloid (THP-1) and lymphoid (Jurkat) leukaemia cell lines when treated with their lowest significant doses (LSDs) that induce apoptosis (determined previously in Mahbub et al., 2013, 2015, 2019) for 24 h. Gene and protein data are expressed as medians and ranges. Results were considered statistically significant when P ≤ 0.05 (*). The combination treatment effects and colours were determined based on the statistical analysis as described in.

In THP-1 acute myeloid cells, the combination treatment of apigenin and etoposide caused a synergistic increase in TNFR1/TNFRSF1A gene and protein expression, when compared to the vehicle controls and drugs alone (P ≤ 0.05) (Fig. 5A,B) (Supplementary Figs. S1, S2). Whilst, when apigenin was used in combination with cyclophosphamide, although a significant increase was seen in the expression of TNFR1/TNFRSF1A compared to vehicle controls (P ≤ 0.05) no difference was seen compared to drug treatments alone (Fig. 5A); and protein expression was unchanged (Fig. 5B) (Supplementary Figs. S1, S2)*.* In THP-1 cells, both combination treatments caused a significant increase in gene and protein expression of TRAILR1/DR4 (Fig. 5) (Supplementary Figs. S1, S2), and gene expression in FAS/CD95 and FADD, when compared to the vehicle control and/or one of the individual drugs, (P ≤ 0.05); with differential effects being seen in protein expression of FAS/CD95, and FADD (Fig. 6) (Supplementary Figs. S1, S2).

In Jurkat acute lymphoid cells, the combination treatment of apigenin and etoposide caused a significant increase in the expression of all investigated death receptors and domain genes and proteins when compared to the vehicle controls (P ≤ 0.05), no synergistic effects were seen (Figs. 5 and 6) (Supplementary Figs. S1, S2). In contrast, the combination treatment of apigenin and cyclophosphamide, caused a synergistic increase in the expression of TRAILR1/DR4 gene and protein levels, when compared to the vehicle controls and drugs alone (P ≤ 0.05) (Fig. 5A,B) (Supplementary Figs. S1, S2). However, this combination did not cause any significant effect on the expression of TNFR1/TNFRSF1A (Fig. 5A,B) FAS/CD95, and FADD (Fig. 6A,B) genes and proteins (Supplementary Figs. S1, S2).

### Effect of combination treatments on expression of caspase genes and proteins (CASP-8, CASP-9, CASP-3)

**Figure 7:**
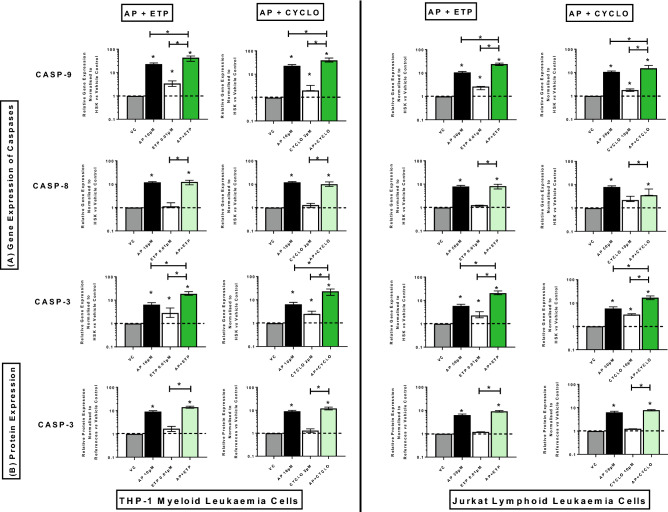
Effects of apigenin (AP) alone and in combination with etoposide (ETP) and cyclophosphamide (CYCLO) on expression of caspase genes (**A**) and proteins (**B**) in acute myeloid (THP-1) and lymphoid (Jurkat) leukaemia cell lines when treated with their lowest significant doses (LSDs) that induce apoptosis (determined previously in Mahbub et al. 2013, 2015, 2019) for 24 h. Gene and protein data are expressed as medians and ranges. Results were considered statistically significant when P ≤ 0.05 (*). The combination treatment effects and colours were determined based on the statistical analysis as described in.

In both myeloid and lymphoid leukaemia cell lines, all combination treatments caused a synergistic increase in CASP-9 and CASP-3 gene expression when compared to the vehicle control and drugs alone (P ≤ 0.05) (Fig. 7A). Whilst CASP-8 was significantly increased compared to the vehicle control and chemotherapy treatments alone (P ≤ 0.05), but not significantly different from that seen from apigenin alone (Fig. 7A). CASP-3 protein expression was also significantly increased in both THP-1 and Jurkat cells when combination treatments were performed compared to the vehicle control and chemotherapy alone (P ≤ 0.05), but no difference was seen from apigenin treatments alone (Fig. 7B) (Supplementary Figs. S1, S2).

## Discussion

Our earlier work has shown that polyphenols can decrease cell proliferation, cause cell cycle arrest, and induce apoptosis in leukaemia cell lines^23,55^. Furthermore, we have shown that the polyphenol, apigenin acts synergistically with chemotherapy agents such as etoposide (a topoisomerase inhibitor) and cyclophosphamide (an alkylating agent), causing a reduction in ATP and glutathione levels, DNA damage and induction of apoptosis in acute myeloid (THP-1) and lymphoid (Jurkat) leukaemia cell lines as shown by induction of caspase activity and morphological evidence of apoptosis^63,64^. To progress our understanding of the therapeutic potential of apigenin, it is essential to elucidate their molecular mechanism of action during apoptosis. Here, we investigated the molecular events that trigger apoptosis in response to apigenin alone, and in combination with etoposide and cyclophosphamide in both acute myeloid (THP-1) and lymphoid (Jurkat) leukaemia cells, following treatment for 24 h.

There are two core pathways involved in inducing apoptosis: (1) The intrinsic or mitochondrial-mediated pathway, and (2) the extrinsic or death receptor-mediated pathway^5,16^. Initiation of either pathway ultimately results in a caspase activation cascade and cellular death^5,16,17^. Here, 8 key genes and proteins of the intrinsic apoptosis pathway (BAD, BAX, BCL2, BCLX, CYT *c*, SMAC/DIABLO, HTRA2/OMI, CASP-9); and 5 genes and proteins of the extrinsic pathway (TRAILR1/DR4, TNFR1/TNFRSF1A, FAS, FADD, CASP-8); plus, the executioner caspase 3 (CASP-3); were significantly modulated by our investigated treatments. Although all responses seen at gene level were not always translated to protein responses; this may be due to the use here of a single time course of 24 h, and changes in the protein levels may take longer, beyond this time point.

This study demonstrated apigenin increased gene and protein expression for BAX, BAD, SMAC/DIABLO and CASP-9, but had no effect on the expression of anti-apoptotic BCL2 and BCLX genes and proteins. In addition, apigenin also induced TRAILR1/DR4, TNFR1/TNFRSF1A, and FAS/CD95 and CASP-8, in both acute leukaemia cell lines. Demonstrating the increase in CASP-3 activity was induced via both intrinsic and extrinsic apoptotic pathways. Apigenin, has previously been shown to induce apoptosis in leukaemia cells (HL60, THP-1, U937) via mitochondrial-dependent mechanisms, with the release of cytochrome *c* to the cytosol and the activation of caspase-9 and -3^51–54^. Similarly, Wang et al*.,* 1999 reported that apigenin could induce apoptosis through cytochrome c release and activation of caspase 9 and 3 in HL60 cells^51^.

Etoposide and cyclophosphamide alone induced apoptosis in both cell lines associated with increased CASP-3, however gene and protein regulation differed between THP-1 and Jurkat cells suggesting differential regulation between myeloid and lymphoid cells. In THP-1 cells, both etoposide and cyclophosphamide reduced anti-apoptotic BCL2 and/or BCLX, and an increase in the expression of TNFR1/TNFRSF1A, and increased CASP-9 and -3. In Jurkat lymphoid cells, etoposide increased BAX, TNFR1/TNFRSF1A, CASP-9, and -3 genes. Whereas cyclophosphamide was much more effective in Jurkat cells and seemed to specifically target intrinsic apoptosis; and increased the gene and protein expression of pro-apoptotic BAD and BAX, CYT *c*, CASP-9 and -3. Indeed, previous studies suggest most chemotherapeutic drugs induce mitochondrial membrane permeabilization prior to apoptosis^65,68^.

**Figure 8:**
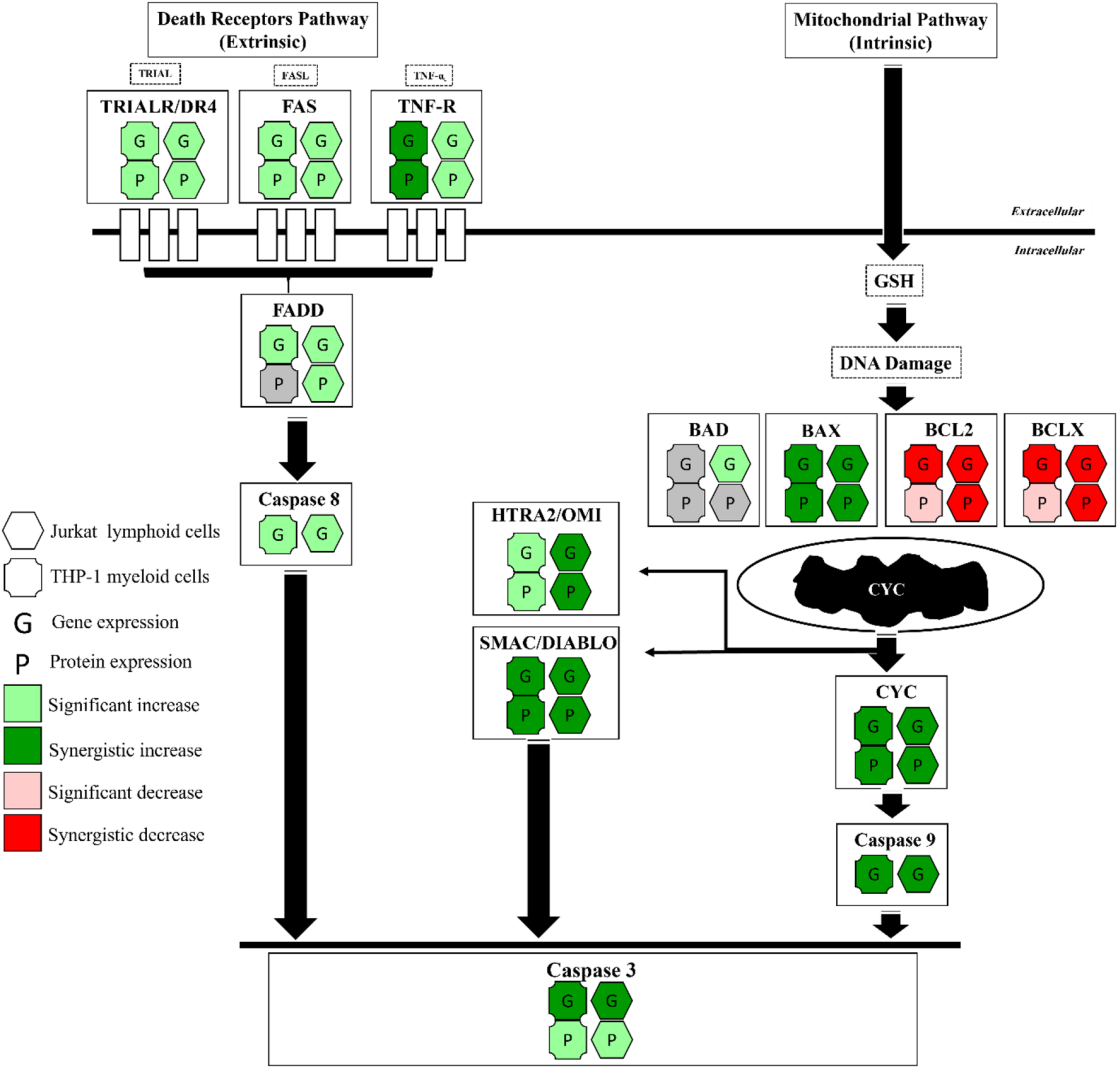
A summary of the effects of apigenin and etoposide combination treatments on apoptosis-related gene and protein expression in acute leukaemia cells (Jurkat and THP-1).

**Figure 9:**
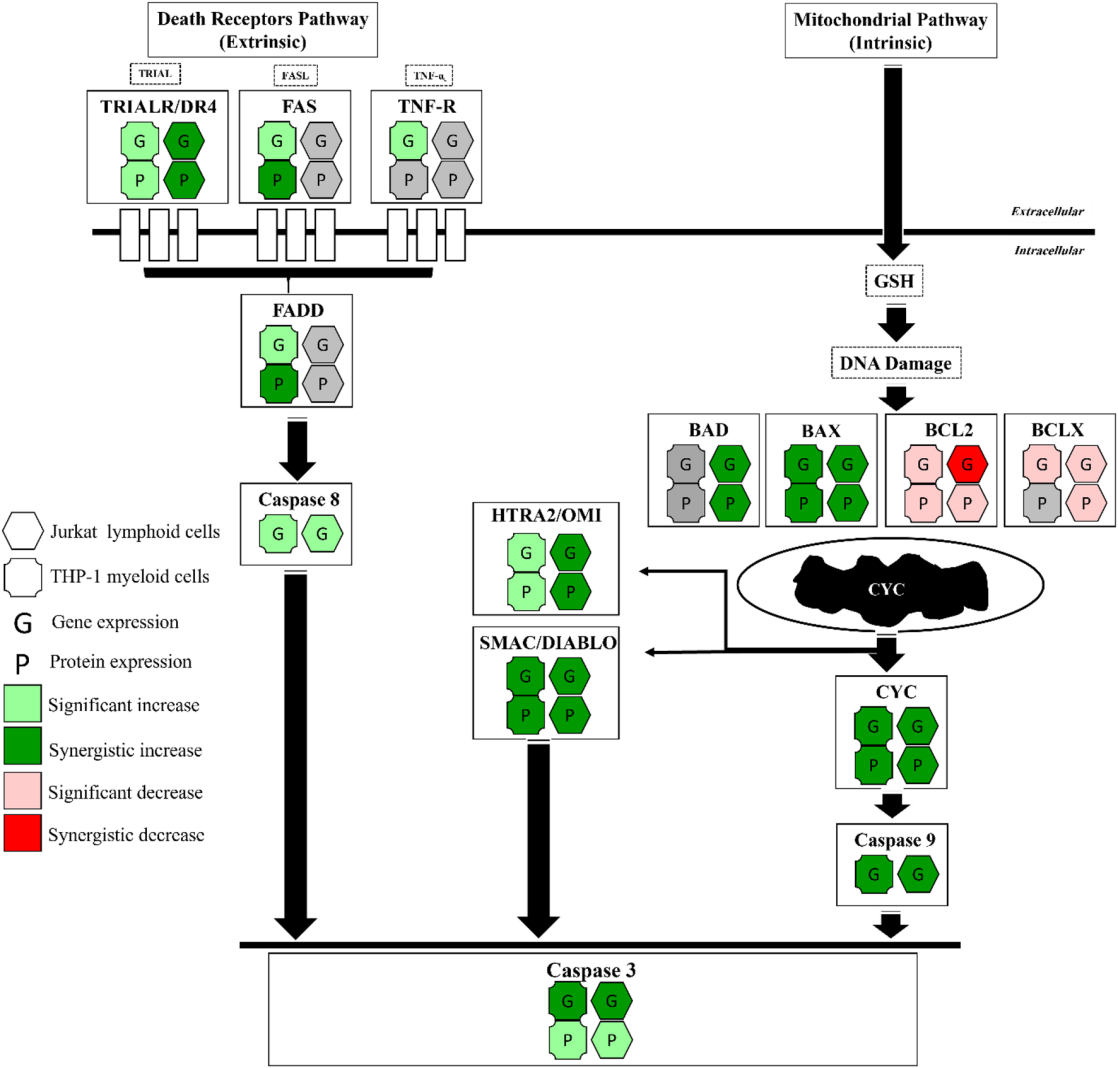
A summary of the effects of apigenin and cyclophosphamide combination treatments on apoptosis-related gene and protein expression in acute leukaemia cells (Jurkat and THP-1).

Combination treatments of apigenin and chemotherapy agents (etoposide or cyclophosphamide), induced apoptosis via a synergistic induction of the intrinsic apoptosis pathway, combined with an apigenin-induced extrinsic apoptosis, resulting in an overall enhanced induction of apoptosis. Specifically, apigenin enhanced the pro-apoptotic activity of chemotherapy agents, through the synergistic increase in expression of BAX, CYT *c*, SMAC/DIABLO, HTRA2/OMI, CASP-9, and -3 gene and protein levels, whilst decreasing anti-apoptotic BCL2 gene expression (Figs. 8 and 9).

Hassanpour et al., 2018 reported that the dysfunction of the intrinsic apoptotic pathway is a key strategy to avoid apoptosis in leukaemia cells^2^. Acute leukaemia is commonly associated with a decrease in cell sensitivity to pro-apoptotic signals due to overexpression of anti-apoptotic proteins of the BCL-2 family such as BCL2 and BCLX^3,9,10,16,69^; and/or with low levels of pro-apoptotic members such as BAX^11^. Yoo et al., 2012 and Jan et al., 2019 reported that targeting the expression of anti-apoptotic and/or pro-apoptotic members of the apoptotic pathways is a promising strategy to induce cancer cell death and improve sensitivity to chemotherapy agents^16,65^. Cassier et al., 2017 also reported that downstream effectors of the mitochondrial pathway are mainly deregulated in acute myeloid leukaemia (AML) through overexpression of anti-apoptotic proteins of BCL2 and BCLX^69^. Overexpression of anti-apoptotic proteins BCL2 and BCLX inhibit apoptosis; and are reported to be correlated with the pathogenesis of acute leukaemia (mainly AML), and can induced resistance to chemotherapeutics^16,17,65^. Here, the effective targeting of anti-apoptotic members BCL2 and BCLX, by the use of apigenin with standard chemotherapy agents; seem to vary dependent on the type of leukaemia cell line and chemotherapy agent used, with apigenin showing a greater modulation of anti-apoptotic members in lymphoid, compared to myeloid cells. Similar reductions in BCLX expression seen here in Jurkat cells have been seen when apigenin was used in combination with TRAIL^70^. Suggesting at least in lymphoid cells apigenin may be able to overcome the overexpression of anti-apoptotic proteins seen during leukaemia.

Notably, both acute leukaemia cells (THP-1 and Jurkat) showed a synergistic increase in gene and protein expression of pro-apoptotic BAX when treated with all combination treatments (Figs. 8 and 9), whilst in Jurkat cells when treated with apigenin and cyclophosphamide, this was accompanied by a synergistic increase of BAD (Fig. 9). In a similar study, when apigenin was used in combination with the BCL2 inhibitor navitoclax for 48 h, there was an upregulation of BAX and BIM gene expression and protein levels, which led to apoptotic cell death in colon cancer (HTC-116) cells^60^. Likewise, Chen et al., 2019 showed that apigenin could inhibit tumorigenesis and induce apoptosis in cisplatin-resistant colon cancer cells (HT-295), when grown in vitro and in vivo as mouse xenografts, through the upregulation of pro-apoptotic BAX and downregulation of anti-apoptotic BCL2^71^.

Conventional chemotherapy agents used clinically, are known to indirectly activate BAX^72^. Liu et al., 2016 and Walensky et al., 2019 reported that the direct activation of BAX, holds great promise for cancer therapies, with the advantages of specificity, and the potential of overcoming chemo- and radio-resistance; and that this is a possible target for small-molecule drug discovery^72,73^. Here, the synergistic increase of BAX gene and protein expression in both THP-1 and Jurkat cells with all combination treatments, was associated with a synergistic increase in CYT *c* and SMAC/DIABLO, and/or HTRA2/OMI (Figs. 8 and 9).

Cytochrome *c* (CYT *c*) is considered to be a critical mediator and biomarker in mitochondria-mediated apoptosis^74–76^. It is located on the inner surface of the mitochondria, it is a heme-containing metalloprotein and multifunctional enzyme, that is involved in cell apoptosis^74^. Functionally, overexpression of cytochrome *c* effectively suppresses the proliferation of cancer cells and induces cell apoptosis, whilst the knockdown of cytochrome c, reverses these effects^74^. SMAC/DIABLO and HTRA2/OMI are important also to neutralize the inhibitory effect of inhibitors of apoptosis proteins (IAPs) on caspase-9 and -3^68^. So, the release of cytochrome *c*, SMAC/DIABLO, and HTRA2/OMI promote the activation of initiator caspase-9 and subsequently the executioner caspases (6, 7 and 3), which leads to apoptotic cell death^68,74^. Here, the gene expression of caspase 9 was synergistically increased in both THP-1 and Jurkat cells when treated with combination treatments of apigenin and etoposide or cyclophosphamide (Figs. 8 and 9), as a result of increased BAX, CYT *c*, and SMAC/DIABLO activation**.** Similarly, three previous studies found that apigenin could synergistically increase caspase 9 gene expression, when used in combination with: (1) TRAIL for 24 h in Jurkat acute lymphoblastic leukaemia cells^70^, (2) Abivertinib for 24 h in U2932 and OVI-LY1 diffuse large B-cell lymphoma cells; via the downregulation of PI3K/mTOR^62^; and (3) Cisplatin in A549 lung cancer cells^61^. In addition, Horinka et al., 2006 and Huang et al., 2020 also reported that apigenin/chemotherapy combination treatments synergistically upregulated caspase 8 activity in Jurkat, U2932, and OCI-LY1 cells^62,70^.

Death receptor related genes and proteins were shown to be increased in this study by apigenin, however these failed to demonstrate synergistic responses when combined with chemotherapy agents, with the only synergistic increases seen in TNFR1/TNFRSF1A in THP-1 myeloid leukaemia cells, when treated with apigenin and etoposide (Fig. 8); and in TRAILR1/DR4 in Jurkat lymphoid leukaemia cells when treated with apigenin and cyclophosphamide (Fig. 9). Apigenin alone was capable of inducing these death receptors which chemotherapy agents were not able to induce alone. Suggesting combined therapies which maintained the effect induced by apigenin alone could enhance apoptosis induction from chemotherapy alone via activation of additional apoptosis pathways. This was further supported by the induction of caspase 8 activity in cells treated with apigenin, but not chemotherapy. Apigenin has previously been shown to target the extrinsic pathway of apoptosis when used in combination with: (1) TRAIL in Jurkat cells via upregulation of the expression of the DR5 gene^70^; and (2) 5-fluorouracil and cisplatin in head and neck carcinoma cells (SCC25 and A431) via upregulation of the expression of TNFR and TRAILR genes^58^.

The synergistic induction of the intrinsic apoptosis pathway, and additional induction of the extrinsic pathway by apigenin resulted in a synergistic increase in caspase 3 activity (Figs. 8 and 9). Caspase 3 has previously been shown to be synergistically increased when apigenin is used in combination with: (1) TRAIL at 24 h for acute lymphoblastic leukaemia cells (Jurkat) via upregulation of DR5 expression^70^, (2) Abivertinib at 24 h for diffuse large B-cell lymphoma cells (U2932 and OCI-LY1) via downregulation of PI3K/mTOR expression^62^; (3) 5-Fluorouracil at 72 h for breast cancer cells (MDA-MB-453) via down regulation of Akt expression^57^; and (4) Cisplatin for lung cancer cells (A549)^61^.

Our previous study showed that polyphenols, including apigenin synergistically enhanced the action of chemotherapeutic agents, including etoposide^63^ and cyclophosphamide^64^ in leukaemia cells (including Jurkat and THP-1), through a reduction of glutathione levels. Furthermore, these studies found that these polyphenol/chemotherapy combination treatments synergistically reduced cell ATP levels, arrested cell cycle, caused DNA damage, and induced apoptosis^63,64^. Interestingly, many researchers reported that there is a strong correlation between glutathione depletion and restored apoptosis induction^75,77^. Franco et al., 2009 reported that a depletion in the glutathione content can directly activate the intrinsic apoptotic pathway, either by: activating the initiator BAX, releasing the cytochrome c from the mitochondria, or by forming the apoptosome; which can be oxidised for its pro-apoptotic action. This may explain why the investigated combination agents here, synergistically increased the expression of mitochondrial BAX and cytochrome *c*. Franco et al., 2009 reported that the depletion of glutathione, restored apoptosis via the intrinsic apoptotic pathway; and suggested that this could be a highly effective way to increase the efficacy of chemotherapy or anti-cancer agents^75,77^. In addition, Traverso et al., 2013 reported that high glutathione levels are commonly found in cancer cells and the efflux of glutathione is one of the major key mechanisms in the development of multi-drug resistance in cancer^77^. Our earlier work has found that the basal glutathione levels of the leukaemia cell lines were linked with the sensitivity to the treatments of polyphenol and chemotherapy^63^. It was demonstrated that Jurkat lymphoid leukaemia cell lines had lower basal glutathione levels than the THP-1 myeloid cell lines and non-tumour control cells^63,64^. This could explain why the Jurkat acute lymphoid cell lines are more sensitive and susceptible.

In conclusion, the combination of apigenin with etoposide and cyclophosphamide induced apoptosis by the synergistic increase of BAX expression (Figs. 8 and 9), the permeabilization of the mitochondrial membrane, and the release of CYT *c*, SMAC/DIABLO, and HTRA2/OMI, which then promotes caspase-9 and -3 activation (Figs. 8 and 9). Furthermore, the induction of the extrinsic apoptosis pathway with induction of death receptors and ligands and caspase 8 by apigenin, activation of which were not seen in chemotherapy stimulations alone could further enhance the total apoptosis seen in combination therapies. Thus, the utilisation of combined therapies targeting apoptosis could have therapeutic potential in the treatment of leukaemia.

## Supplementary Information

Supplementary Information.
