# Supplementary material for: The effect of apigenin and chemotherapy combination treatments on apoptosis-related genes and proteins in acute leukaemia cell lines
Source: Sci Rep. 2022 May 25;12:8858. doi: 10.1038/s41598-022-11441-z (PMC9132959; doi:10.1038/s41598-022-11441-z)
Supplement: Supplementary file 1 — Supplementary Information. [file 41598_2022_11441_MOESM1_ESM.docx]

**Supplementary Materials**

| Supplementary Table 1**: Apoptosis Related Genes List**  **of the TaqMan® Array Human Cellular Apoptosis** | | | |
| --- | --- | --- | --- |
| **#** | **List of Apoptotic Genes** | **#** | **List of Apoptotic Genes** |
| **1** | AIFM1 | **47** | NFKB2 |
| **2** | AKT1 | **48** | NFKBIA |
| **3** | APAF1 | **49** | NFKBIB |
| **4** | ATM | **50** | NFKBIE |
| **5** | BAD | **51** | P53AIP1 |
| **6** | BAK1 | **52** | PARP1 |
| **7** | BAX | **53** | PARP2 |
| **8** | BBC3 | **54** | PARP3 |
| **9** | BCL2 | **55** | PARP4 |
| **10** | BCL2L1/ BCLX | **56** | PIK3CA |
| **11** | BCL2L11 | **57** | PIK3CB |
| **12** | BID | **58** | PIK3CD |
| **13** | BIRC2 | **59** | PIK3R1 |
| **14** | CASP3 | **60** | PIK3R2 |
| **15** | CASP7 | **61** | PMAIP1 |
| **16** | CASP8 | **62** | PPID |
| **17** | CASP9 | **63** | PRKCA |
| **18** | CDKN2A | **64** | PRKCB |
| **19** | CFLAR | **65** | PRKCD |
| **20** | CHEK2 | **66** | PRKCE |
| **21** | CHUK | **67** | PRKCZ |
| **22** | CYT C | **68** | REL |
| **23** | DAPK3 | **69** | RELA |
| **24** | DAXX | **70** | RELB |
| **25** | DFFA | **71** | RIPK1 |
| **26** | DFFB | **72** | RPS6KA1 |
| **27** | SMAC/DIABLO | **73** | RPS6KA2 |
| **28** | EGFR | **74** | RPS6KA3 |
| **29** | ENDOG | **75** | RPS6KA4 |
| **30** | F2RL3 | **76** | RPS6KA5 |
| **31** | FADD | **77** | SLC25A4 |
| **32** | FAS/CD95 | **78** | SLC25A5 |
| **33** | FASLG | **79** | SLC25A6 |
| **34** | HRTA2/OMI | **80** | TGFB1 |
| **35** | IGF1 | **81** | TNF |
| **36** | IGF1R | **82** | TNFRSF10A/ TRAIL R1/DR4/ |
| **37** | IKBKB | **83** | TNFR1/TNFRSF1A |
| **38** | IKBKE | **84** | TNFSF10 |
| **39** | IKBKG | **85** | TNFSF12 |
| **40** | IL2 | **86** | TP53 |
| **41** | IL6 | **87** | TRADD |
| **42** | KDR | **88** | TRAF2 |
| **43** | KIT | **89** | TSPO |
| **44** | MDM2 | **90** | VDAC1 |
| **45** | MET | **91** | VDAC2 |
| **46** | NFKB1 | **92** | VDAC3 |

| Supplementary Table 2**: Apoptosis Related Protein List**  **of the Proteome Profiler^TM^ Human Apoptosis Array** | | | |
| --- | --- | --- | --- |
| **#** | **List of Apoptotic Proteins** | **#** | **List of Apoptotic Proteins** |
| **1** | BAD | **19** | HO-2/HMOX-2 |
| **2** | BAX | **20** | HSP27 |
| **3** | BCL2 | **21** | HSP60 |
| **4** | BCLX | **22** | HSP70 |
| **5** | Pro-CASP3 | **23** | HRTA2/OMI |
| **6** | Cleaved-CASP3 | **24** | Livin |
| **7** | Catalase | **25** | PON2 |
| **8** | cIAP-1 | **26** | P21/CIP1/CDKNA1 |
| **9** | cIAP-2 | **27** | P27/Kip1 |
| **10** | Claspin | **28** | Phospho-P53 (S15) |
| **11** | Clusterin | **29** | Phospho-P53 (S46) |
| **12** | Cytochrom C | **30** | Phospho-P53 (S392) |
| **13** | TRAIL R1/DR4 | **31** | Phospho-P53 (S635) |
| **14** | TRAIL R1/DR5 | **32** | SMAC/DIABLO |
| **15** | FADD | **33** | Survivin |
| **16** | FAS/CD95 | **34** | TNFR1/TNFRSF1A |
| **17** | HIF-1α | **35** | XIAP |
| **18** | HO-1/HMOX-1 | **−** | − |

| Supplementary Table 3**:**  **The Statistical Analysis of AP and ETP Alone and in Combination Treatments**  **in THP-1 Myeloid Leukaemia Cells (P ≤ 0.05)** | | | | | | |
| --- | --- | --- | --- | --- | --- | --- |
| **Apoptotic**  **Genes and Proteins** | | **Treatment alone** | **Treatment alone** | **Combination Treatment (AP+ETP)** | | |
|  |  | **AP 10µM**  ***vs* VC** | **ETP 0.01µM**  ***vs* VC** | **AP+ETP**  ***vs* VC** | **AP+ETP**  ***vs* AP 10µM** | **AP+ETP**  ***vs* ETP 0.01µM** |
| **BCL2** | **Gene** | Not significant  P = 0.0563 | Significant  P = 0.0063 | Significant  P < 0.0001 | Significant  P < 0.0001 | Significant  P = 0.0063 |
|  | **Protein** | Not significant  P = 0.0574 | Significant  P = 0.0474 | Significant  P = 0.0048 | Significant  P = 0.0011 | Not significant  P = 0.0574 |
| **BCLX** | **Gene** | Not significant  P = 0.0616 | Not significant  P = 0.0563 | Significant  P = 0.0007 | Significant  P < 0.0001 | Not significant  P = 0.0578 |
|  | **Protein** | Not significant  P = 0.0648 | Not significant  P = 0.0574 | Not significant  P = 0.0574 | Significant  P = 0.0011 | Not significant  P = 0.0548 |
| **BAD** | **Gene** | Significant  P < 0.0001 | Not significant  P = 0.0633 | Not significant  P = 0.0567 | Significant  P = 0.0133 | Not significant  P = 0.0578 |
|  | **Protein** | Significant  P = 0.0048 | Not significant  P = 0.0554 | Not significant  P = 0.0574 | Significant  P = 0.0474 | Significant  P = 0.0048 |
| **BAX** | **Gene** | Significant  P < 0.0001 | Not significant  P = 0.0563 | Significant  P < 0.0001 | Significant  P = 0.0063 | Significant  P < 0.0001 |
|  | **Protein** | Significant  P = 0.0075 | Not significant  P > 0.9999 | Significant  P = 0.0151 | Significant  P = 0.0278 | Significant  P = 0.0151 |
| **CYT C** | **Gene** | Significant  P < 0.0001 | Not significant  P = 0.0603 | Significant  P < 0.0001 | Significant  P = 0.0063 | Significant  P < 0.0001 |
|  | **Protein** | Significant  P = 0.0048 | Not significant  P = 0.0574 | Significant  P = 0.0011 | Significant  P = 0.0474 | Significant  P = 0.0048 |
| **HTRA2/OMI** | **Gene** | Significant  P = 0.0117 | Not significant  P = 0.0546 | Significant  P = 0.0081 | Not significant  P = 0.8089 | Significant  P = 0.0004 |
|  | **Protein** | Significant  P = 0.0105 | Not significant  P = 0.1778 | Significant  P = 0.0075 | Not significant  P > 0.9999 | Significant  P = 0.0151 |
| **SMAC/DIABLO** | **Gene** | Significant  P = 0.0063 | Not significant  P = 0.0560 | Significant  P < 0.0001 | Significant  P = 0.0063 | Significant  P < 0.0001 |
|  | **Protein** | Significant  P = 0.0474 | Not significant  P = 0.0673 | Significant  P = 0.0048 | Significant  P = 0.0474 | Significant  P = 0.0011 |
| **TRAILR1/DR4** | **Gene** | Significant  P = 0.0003 | Not significant  P = 0.0616 | Significant  P < 0.0001 | Not significant  P = 0.0578 | Significant  P = 0.0007 |
|  | **Protein** | Significant  P = 0.0011 | Not significant  P = 0.0544 | Significant  P = 0.0048 | Significant  P = 0.0474 | Not significant  P = 0.0574 |
| **TNFR1/TNFRSF1A** | **Gene** | Significant  P < 0.0001 | Significant  P = 0.0063 | Significant  P < 0.0001 | Significant  P = 0.0063 | Significant  P < 0.0001 |
|  | **Protein** | Significant  P = 0.0474 | Not significant  P = 0.0564 | Significant  P = 0.0048 | Significant  P = 0.0474 | Significant  P = 0.0011 |
| **FAS/CD95** | **Gene** | Significant  P = 0.0126 | Not significant  P = 0.5651 | Significant  P = 0.0094 | Not significant  P = 0.8465 | Significant  P = 0.0039 |
|  | **Protein** | Significant  P = 0.0048 | Not significant  P = 0.0574 | Significant  P = 0.0011 | Not significant  P = 0.0574 | Significant  P = 0.0048 |
| **FADD** | **Gene** | Significant  P = 0.0006 | Not significant  P = 0.0546 | Significant  P = 0.0004 | Not significant  P = 0.8089 | Significant  P = 0.0081 |
|  | **Protein** | Significant  P = 0.0011 | Not significant  P = 0.0568 | Not significant  P = 0.0578 | Significant  P = 0.0474 | Not significant  P = 0.0574 |
| **CASP-8** | **Gene** | Significant  P = 0.0052 | Not significant  P = 0.5651 | Significant  P = 0.0039 | Not significant  P = 0.8465 | Significant  P = 0.0094 |
| **CASP-9** | **Gene** | Significant  P < 0.0001 | Significant  P = 0.0063 | Significant  P < 0.0001 | Significant  P = 0.0063 | Significant  P < 0.0001 |
| **CASP-3** | **Gene** | Significant  P < 0.0001 | Significant  P = 0.0063 | Significant  P < 0.0001 | Significant  P = 0.0063 | Significant  P < 0.0001 |
|  | **Protein** | Significant  P = 0.0048 | Not significant  P = 0.0574 | Significant  P = 0.0011 | Not significant  P = 0.0604 | Significant  P = 0.0048 |
| **Colour Key:** Black shows no significant change, green shows a significant increase, red shows a significant decrease in expression of apoptotic genes/proteins. | | | | | | |

| Supplementary Table 4**:**  **The Statistical Analysis of AP and CYCLO Alone and in Combination Treatments**  **in THP-1 Myeloid Leukaemia Cells (P ≤ 0.05)** | | | | | | |
| --- | --- | --- | --- | --- | --- | --- |
| **Apoptotic**  **Genes and Proteins** | | **Treatment alone** | **Treatment alone** | **Combination Treatment (AP+CYCLO)** | | |
|  |  | **AP 10µM**  ***vs* VC** | **CYCLO 2µM**  ***vs* VC** | **AP+CYCLO**  ***vs* VC** | **AP+CYCLO**  ***vs* AP 10µM** | **AP+CYCLO**  ***vs* CYCLO 2µM** |
| **BCL2** | **Gene** | Not significant  P = 0.0563 | Significant  P = 0.0063 | Significant  P < 0.0001 | Significant  P < 0.0001 | Not significant  P = 0.0563 |
|  | **Protein** | Not significant  P = 0.0574 | Significant  P = 0.0474 | Significant  P = 0.0048 | Significant  P = 0.0011 | Not significant  P = 0.0574 |
| **BCLX** | **Gene** | Not significant  P = 0.0616 | Significant  P = 0.0147 | Significant  P = 0.0047 | Significant  P = 0.0003 | Not significant  P = 0.4609 |
|  | **Protein** | Not significant  P = 0.0648 | Significant  P = 0.0048 | Not significant  P = 0.0584 | Significant  P = 0.0048 | Not significant  P = 0.0574 |
| **BAD** | **Gene** | Significant  P < 0.0001 | Significant  P = 0.0063 | Not significant  P = 0.0563 | Significant  P = 0.0063 | Significant  P < 0.0001 |
|  | **Protein** | Significant  P = 0.0048 | Significant  P = 0.0474 | Not significant  P = 0.0574 | Significant  P = 0.0474 | Significant  P = 0.0048 |
| **BAX** | **Gene** | Significant  P < 0.0001 | Not significant  P = 0.4609 | Significant  P = 0.0006 | Significant  P = 0.0486 | Significant  P = 0.0003 |
|  | **Protein** | Significant  P = 0.0075 | Not significant  P > 0.9999 | Significant  P = 0.0151 | Significant  P = 0.0178 | Significant  P = 0.0151 |
| **CYT C** | **Gene** | Significant  P < 0.0001 | Not significant  P = 0.4609 | Significant  P = 0.0003 | Significant  P = 0.0486 | Significant  P = 0.0006 |
|  | **Protein** | Significant  P = 0.0048 | Not significant  P = 0.0574 | Significant  P = 0.0011 | Significant  P = 0.0474 | Significant  P = 0.0048 |
| **HTRA2/OMI** | **Gene** | Significant  P = 0.0117 | Not significant  P = 0.5108 | Significant  P = 0.0018 | Not significant  P = 0.147 | Significant  P = 0.0007 |
|  | **Protein** | Significant  P = 0.0105 | Not significant  P = 0.0574 | Significant  P = 0.0048 | Not significant  P = 0.0567 | Significant  P = 0.0011 |
| **SMAC/DIABLO** | **Gene** | Significant  P = 0.0063 | Not significant  P = 0.0563 | Significant  P < 0.0001 | Significant  P = 0.0063 | Significant  P < 0.0001 |
|  | **Protein** | Significant  P = 0.0474 | Not significant  P = 0.0574 | Significant  P = 0.0048 | Significant  P = 0.0474 | Significant  P = 0.0011 |
| **TRAILR1/DR4** | **Gene** | Significant  P = 0.0003 | Not significant  P = 0.0586 | Significant  P = 0.0047 | Not significant  P = 0.4609 | Significant  P = 0.0003 |
|  | **Protein** | Significant  P = 0.0011 | Not significant  P = 0.0573 | Significant  P = 0.0048 | Significant  P = 0.0048 | Significant  P = 0.0474 |
| **TNFR1/TNFRSF1A** | **Gene** | Significant  P < 0.0001 | Significant  P = 0.0033 | Significant  P = 0.0092 | Not significant  P = 0.2441 | Not significant  P = 0.4929 |
|  | **Protein** | Significant  P = 0.0474 | Not significant  P = 0.145 | Not significant  P = 0.145 | Not significant  P = 0.4818 | Not significant  P > 0.9999 |
| **FAS/CD95** | **Gene** | Significant  P = 0.0126 | Not significant  P = 0.0563 | Significant  P < 0.0001 | Not significant  P = 0.0563 | Significant  P < 0.0001 |
|  | **Protein** | Significant  P = 0.0048 | Not significant  P = 0.0574 | Significant  P = 0.0011 | Significant  P = 0.0474 | Significant  P = 0.0048 |
| **FADD** | **Gene** | Significant  P = 0.0006 | Not significant  P = 0.0563 | Significant  P < 0.0001 | Not significant  P = 0.0563 | Significant  P < 0.0001 |
|  | **Protein** | Significant  P = 0.0011 | Not significant  P = 0.0474 | Significant  P = 0.0048 | Significant  P = 0.0048 | Significant  P = 0.0011 |
| **CASP-8** | **Gene** | Significant  P = 0.0052 | Not significant  P = 0.0572 | Significant  P = 0.0005 | Not significant  P = 0.2029 | Significant  P = 0.0158 |
| **CASP-9** | **Gene** | Significant  P < 0.0001 | Significant  P = 0.0063 | Significant  P < 0.0001 | Significant  P = 0.0063 | Significant  P < 0.0001 |
| **CASP-3** | **Gene** | Significant  P < 0.0001 | Significant  P = 0.0063 | Significant  P < 0.0001 | Significant  P = 0.0063 | Significant  P < 0.0001 |
|  | **Protein** | Significant  P = 0.0048 | Not significant  P = 0.0574 | Significant  P = 0.0011 | Not significant  P = 0.0574 | Significant  P = 0.0048 |
| **Colour Key:** Black shows no significant change, green shows a significant increase, red shows a significant decrease in expression of apoptotic genes/proteins. | | | | | | |

| Supplementary Table 5**:**  **The Statistical Analysis of AP and ETP Alone and in Combination Treatments**  **in Jurkat Lymphoid Leukaemia Cells (P ≤ 0.05)** | | | | | | |
| --- | --- | --- | --- | --- | --- | --- |
| **Apoptotic**  **Genes and Proteins** | | **Treatment alone** | **Treatment alone** | **Combination Treatment (AP+ETP)** | | |
|  |  | **AP 50µM**  ***vs* VC** | **ETP 0.01µM**  ***vs* VC** | **AP+ETP**  ***vs* VC** | **AP+ETP**  ***vs* AP 50µM** | **AP+ETP**  ***vs* ETP 0.01µM** |
| **BCL2** | **Gene** | Not significant  P = 0.7109 | Not significant  P = 0.8067 | Significant  P = 0.0476 | Significant  P = 0.0476 | Significant  P = 0.0445 |
|  | **Protein** | Not significant  P = 0.4474 | Not significant  P = 0.1776 | Significant  P = 0.0048 | Significant  P = 0.0011 | Not significant  P = 0.0574 |
| **BCLX** | **Gene** | Not significant  P = 0.4609 | Not significant  P = 0.4676 | Significant  P = 0.0115 | Significant  P = 0.02 | Significant  P = 0.0158 |
|  | **Protein** | Not significant  P > 0.9999 | Not significant  P = 0.36 | Significant  P = 0.0011 | Significant  P = 0.0048 | Significant  P = 0.0474 |
| **BAD** | **Gene** | Significant  P = 0.0081 | Not significant  P = 0.5403 | Significant  P = 0.0037 | Not significant  P = 0.3175 | Significant  P = 0.0016 |
|  | **Protein** | Significant  P = 0.0474 | Not significant  P = 0.0674 | Not significant  P = 0.1011 | Not significant  P = 0.0773 | Significant  P = 0.0048 |
| **BAX** | **Gene** | Significant  P = 0.0047 | Significant  P = 0.0147 | Significant  P = 0.0001 | Significant  P = 0.0147 | Significant  P = 0.0047 |
|  | **Protein** | Significant  P = 0.0474 | Not significant  P = 0.0774 | Significant  P = 0.0011 | Significant  P = 0.0474 | Significant  P = 0.0048 |
| **CYT C** | **Gene** | Not significant  P = 0.0663 | Not significant  P = 0.1573 | Significant  P = 0.0001 | Significant  P = 0.0047 | Significant  P = 0.0147 |
|  | **Protein** | Not significant  P = 0.0774 | Not significant  P = 0.0705 | Significant  P = 0.005 | Significant  P = 0.008 | Significant  P = 0.008 |
| **HTRA2/OMI** | **Gene** | Not significant  P = 0.647 | Not significant  P = 0.8089 | Significant  P = 0.0001 | Significant  P = 0.0117 | Significant  P = 0.0081 |
|  | **Protein** | Not significant  P = 0.0674 | Not significant  P = 0.0594 | Significant  P = 0.0011 | Significant  P = 0.0474 | Significant  P = 0.0048 |
| **SMAC/DIABLO** | **Gene** | Significant  P = 0.0063 | Not significant  P = 0.4609 | Significant  P = 0.0006 | Significant  P = 0.0486 | Significant  P = 0.0003 |
|  | **Protein** | Significant  P < 0.0001 | Not significant  P = 0.0674 | Significant  P = 0.0011 | Significant  P = 0.0474 | Significant  P = 0.0048 |
| **TRAILR1/DR4** | **Gene** | Significant  P < 0.0001 | Not significant  P = 0.0616 | Significant  P < 0.0001 | Not significant  P = 0.0578 | Significant  P = 0.0007 |
|  | **Protein** | Significant  P = 0.0474 | Not significant  P > 0.9999 | Significant  P = 0.0151 | Not significant  P = 0.1778 | Significant  P = 0.0151 |
| **TNFR1/TNFRSF1A** | **Gene** | Significant  P < 0.0001 | Significant  P = 0.0117 | Significant  P = 0.0001 | Not significant  P = 0.0617 | Not significant  P = 0.0681 |
|  | **Protein** | Significant  P = 0.0151 | Not significant  P = 0.0574 | Significant  P = 0.0048 | Not significant  P = 0.0604 | Significant  P = 0.0011 |
| **FAS/CD95** | **Gene** | Significant  P = 0.0006 | Not significant  P = 0.0633 | Significant  P < 0.0001 | Not significant  P = 0.0578 | Not significant  P = 0.0633 |
|  | **Protein** | Significant  P = 0.008 | Not significant  P = 0.0574 | Significant  P = 0.0474 | Significant  P = 0.0474 | Significant  P = 0.0048 |
| **FADD** | **Gene** | Not significant  P = 0.1778 | Not significant  P = 0.0581 | Significant  P = 0.0001 | Not significant  P = 0.0577 | Significant  P = 0.0481 |
|  | **Protein** | Not significant  P = 0.1324 | Not significant  P = 0.0574 | Significant  P = 0.0048 | Not significant  P = 0.0674 | Significant  P = 0.0011 |
| **CASP-8** | **Gene** | Significant  P < 0.0001 | Not significant  P = 0.0546 | Significant  P = 0.0004 | Not significant  P = 0.8089 | Significant  P = 0.0081 |
| **CASP-9** | **Gene** | Significant  P < 0.0001 | Significant  P = 0.0063 | Significant  P < 0.0001 | Significant  P = 0.0063 | Significant  P < 0.0001 |
| **CASP-3** | **Gene** | Significant  P < 0.0001 | Significant  P = 0.0063 | Significant  P < 0.0001 | Significant  P = 0.0063 | Significant  P < 0.0001 |
|  | **Protein** | Significant  P = 0.0048 | Not significant  P = 0.0574 | Significant  P = 0.0011 | Not significant  P = 0.0544 | Significant  P = 0.0048 |
| **Colour Key:** Black shows no significant change, green shows a significant increase, red shows a significant decrease in expression of apoptotic genes/proteins. | | | | | | |

| Supplementary Table 6**:**  **The Statistical Analysis of AP and CYCLO Alone and in Combination Treatments**  **in Jurkat Lymphoid Leukaemia Cells (P ≤ 0.05)** | | | | | | |
| --- | --- | --- | --- | --- | --- | --- |
| **Apoptotic**  **Genes and Proteins** | | **Treatment alone** | **Treatment alone** | **Combination Treatment (AP+CYCLO)** | | |
|  |  | **AP 50µM**  ***vs* VC** | **CYCLO 10µM**  ***vs* VC** | **AP+CYCLO**  ***vs* VC** | **AP+CYCLO**  ***vs* AP 50µM** | **AP+CYCLO**  ***vs* CYCLO 10µM** |
| **BCL2** | **Gene** | Not significant  P = 0.7109 | Not significant  P = 0.6224 | Significant  P = 0.0046 | Significant  P = 0.0047 | Significant  P = 0.0048 |
|  | **Protein** | Not significant  P = 0.4474 | Not significant  P = 0.4474 | Significant  P = 0.0548 | Significant  P = 0.0474 | Not significant  P = 0.0611 |
| **BCLX** | **Gene** | Not significant  P = 0.4609 | Not significant  P = 0.0647 | Significant  P = 0.0147 | Not significant  P = 0.0647 | Significant  P = 0.0001 |
|  | **Protein** | Not significant  P > 0.9999 | Not significant  P = 0.0705 | Significant  P = 0.0151 | Significant  P = 0.0151 | Not significant  P = 0.1778 |
| **BAD** | **Gene** | Significant  P = 0.0081 | Significant  P = 0.0117 | Significant  P = 0.0001 | Significant  P = 0.0117 | Significant  P = 0.0081 |
|  | **Protein** | Significant  P = 0.0474 | Significant  P = 0.0048 | Significant  P = 0.0011 | Significant  P = 0.0048 | Significant  P = 0.0474 |
| **BAX** | **Gene** | Significant  P = 0.0047 | Significant  P=0.0147 | Significant  P = 0.0001 | Significant  P=0.0147 | Significant  P=0.0047 |
|  | **Protein** | Significant  P = 0.0474 | Significant  P = 0.0048 | Significant  P = 0.0011 | Significant  P = 0.0048 | Significant  P = 0.0474 |
| **CYT C** | **Gene** | Not significant  P = 0.0663 | Significant  P < 0.0001 | Significant  P < 0.0001 | Significant  P < 0.0001 | Significant  P = 0.0063 |
|  | **Protein** | Not significant  P = 0.0774 | Significant  P = 0.0048 | Significant  P = 0.0011 | Significant  P = 0.0048 | Significant  P = 0.0474 |
| **HTRA2/OMI** | **Gene** | Not significant  P = 0.647 | Not significant  P = 0.0647 | Significant  P = 0.0001 | Significant  P = 0.0147 | Significant  P = 0.0047 |
|  | **Protein** | Not significant  P = 0.0674 | Not significant  P = 0.0600 | Significant  P = 0.0048 | Significant  P = 0.0474 | Significant  P = 0.0011 |
| **SMAC/DIABLO** | **Gene** | Significant  P = 0.0063 | Not significant  P = 0.0705 | Significant  P < 0.0001 | Significant  P = 0.0063 | Significant  P < 0.0001 |
|  | **Protein** | Significant  P < 0.0001 | Not significant  P = 0.1778 | Significant  P = 0.0151 | Significant  P = 0.0045 | Significant  P = 0.0151 |
| **TRAILR1/DR4** | **Gene** | Significant  P < 0.0001 | Significant  P = 0.0063 | Significant  P < 0.0001 | Significant  P = 0.0063 | Significant  P < 0.0001 |
|  | **Protein** | Significant  P = 0.0474 | Significant  P = 0.0048 | Significant  P = 0.0011 | Significant  P = 0.0048 | Significant  P = 0.0474 |
| **TNFR1/TNFRSF1A** | **Gene** | Significant  P < 0.0001 | Not significant  P = 0.0663 | Not significant  P =0.0601 | Not significant  P = 0.0663 | Not significant  P = 0.0666 |
|  | **Protein** | Significant  P = 0.0151 | Not significant  P > 0.9999 | Not significant  P = 0.0705 | Not significant  P = 0.1778 | Not significant  P = 0.0705 |
| **FAS/CD95** | **Gene** | Significant  P = 0.0006 | Not significant  P = 0.0747 | Not significant  P = 0.4609 | Significant  P = 0.0563 | Not significant  P = 0.0647 |
|  | **Protein** | Significant  P = 0.008 | Not significant  P = 0.0705 | Not significant  P = 0.0754 | Significant  P = 0.0458 | Not significant  P > 0.9999 |
| **FADD** | **Gene** | Not significant  P = 0.1778 | Not significant  P = 0.0705 | Not significant  P = 0.0674 | Not significant  P = 0.0774 | Not significant  P = 0.0664 |
|  | **Protein** | Not significant  P = 0.1324 | Not significant  P > 0.9999 | Not significant  P > 0.9999 | Not significant  P = 0.1324 | Not significant  P > 0.9999 |
| **CASP-8** | **Gene** | Significant  P < 0.0001 | Not significant  P = 0.0663 | Significant  P < 0.0001 | Significant  P = 0.0063 | Not significant  P = 0.0600 |
| **CASP-9** | **Gene** | Significant  P < 0.0001 | Significant  P = 0.0063 | Significant  P < 0.0001 | Significant  P = 0.0063 | Significant  P < 0.0001 |
| **CASP-3** | **Gene** | Significant  P < 0.0001 | Significant  P = 0.0063 | Significant  P < 0.0001 | Significant  P = 0.0063 | Significant  P < 0.0001 |
|  | **Protein** | Significant  P = 0.0048 | Not significant  P = 0.0604 | Significant  P = 0.0011 | Not significant  P = 0.0604 | Significant  P = 0.0048 |
| **Colour Key:** Black shows no significant change, green shows a significant increase, red shows a significant decrease in expression of apoptotic genes/proteins. | | | | | | |


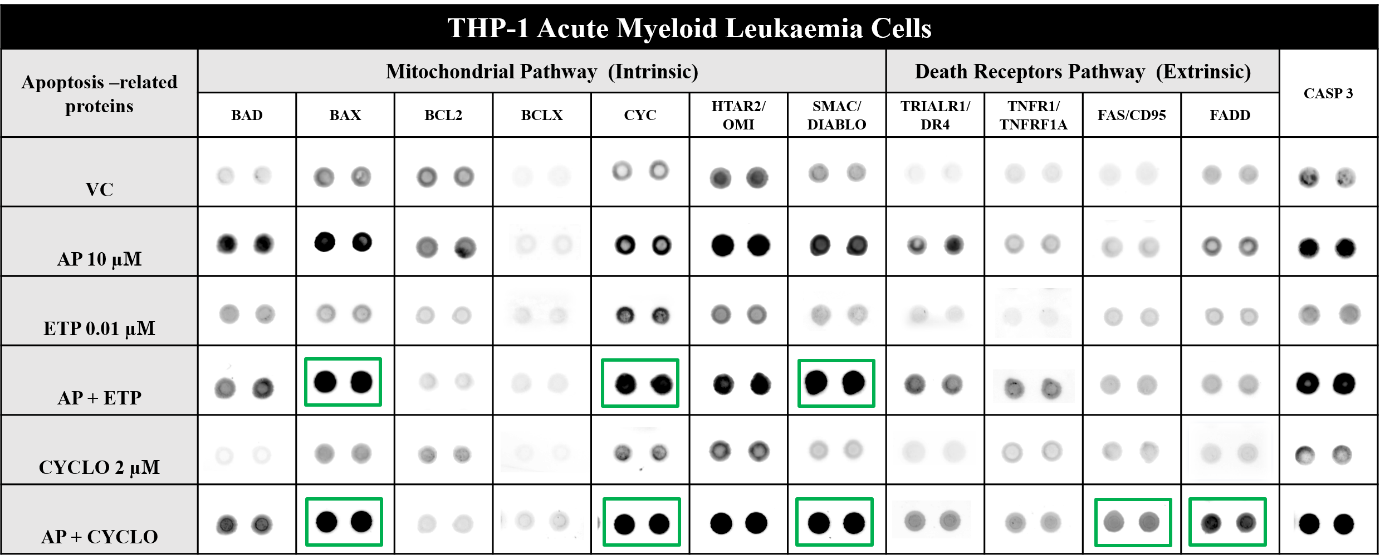


Supplementary Figure 1**: The array data of** apoptosis-related proteins **(BAD, BAX, BCL2, BCLX, CYT *c*, SMAC/ DIBALO, HTRA2/OMI, TRAILR1/DR4, TNFR1/TNFRSF1A, FAS/D95, FADD, and CASP-3)** in THP-1 acute myeloid leukaemia cells treated with apigenin (AP) alone, and in combination **with etoposide (ETP) and cyclophosphamide (CYCLO) at the lowest significant doses (LSDs) that induce apoptosis (determined previously in Mahbub *et al*., 2013; 2015; 2019) for 24 h.** Protein expression was analysed using a Proteome Profiler^TM^ - Human Apoptosis Array (R & D Systems). The combination effect is highlighted in a dark green box (if there is a synergistic increase) and in the dark box (if there is a synergistic decrease).


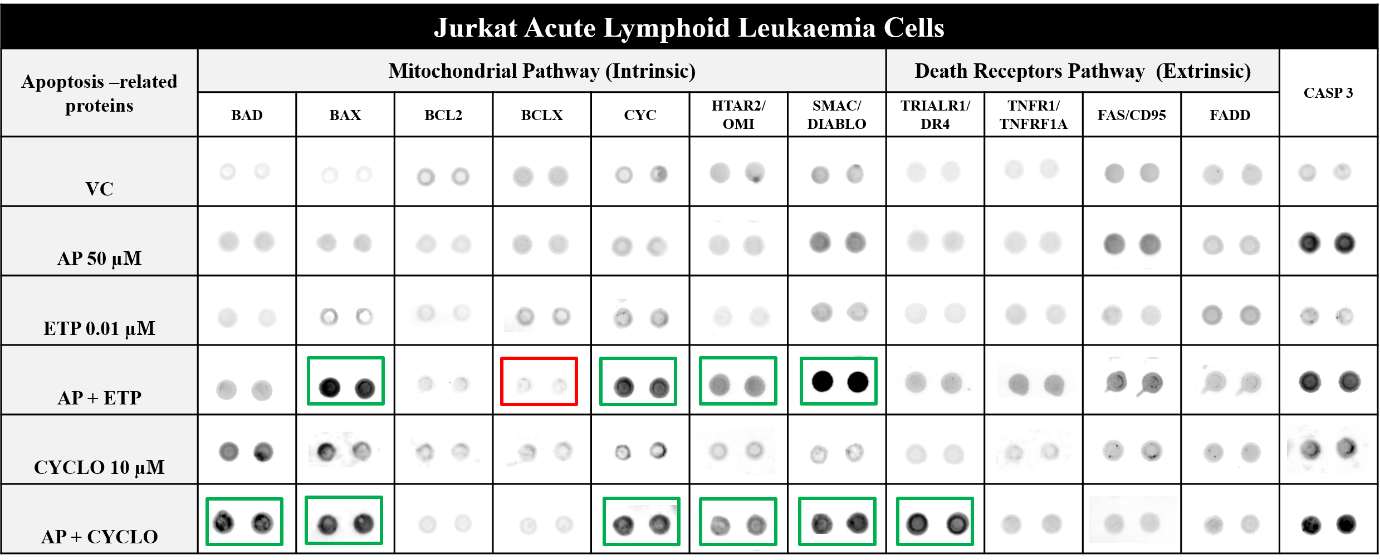


Supplementary Figure 2**: The array data of** apoptosis-related proteins **(BAD, BAX, BCL2, BCLX, CYT *c*, SMAC/ DIBALO, HTRA2/OMI, TRAILR1/DR4, TNFR1/TNFRSF1A, FAS/D95, FADD, and CASP-3)** in Jurkat acute lymphoid leukaemia cells treated with apigenin (AP) alone, and in combination **with etoposide (ETP) and cyclophosphamide (CYCLO) at the lowest significant doses (LSDs) that induce apoptosis (determined previously in Mahbub *et al*., 2013; 2015; 2019) for 24 h.** Protein expression was analysed using a Proteome Profiler^TM^ - Human Apoptosis Array (R & D Systems). The combination effect is highlighted in a dark green box (if there is a synergistic increase) and in the dark box (if there is a synergistic decrease).
